# Modulation of *Streptococcus mutans* Adherence to Hydroxyapatite by Engineered Salivary Peptides

**DOI:** 10.3390/microorganisms10020223

**Published:** 2022-01-20

**Authors:** Lina Maria Marin, Yizhi Xiao, Jaime Aparecido Cury, Walter Luiz Siqueira

**Affiliations:** 1 College of Dentistry, University of Saskatchewan, Saskatoon, SK S7N 5E4, Canada; lina.marin@usask.ca; 2 Schulich School of Dentistry, The University of Western Ontario, London, ON N6A 5C1, Canada; yxiao32@uwo.ca; 3 Piracicaba Dental School, University of Campinas, Piracicaba CEP 13414-903, Brazil; jcury@unicamp.br

**Keywords:** salivary peptides, acquired enamel pellicle, adherence, statherin, histatin, proteomics

## Abstract

Since the modification of the proteinaceous components of the Acquired Enamel Pellicle (AEP) could influence the adhesion of *Streptococcus mutans,* the most cariogenic bacteria, to dental surfaces, we assessed if engineered salivary peptides would affect the adherence and modulate the bacterial proteome upon adherence. Single-component AEPs were formed onto hydroxyapatite (HAp) discs by incubating them with statherin, histatin-3, DR9, DR9-DR9, DR9-RR14, RR14, and parotid saliva. Then, the discs were inoculated with *S. mutans* UA159 and the bacteria were allowed to adhere for 2 h, 4 h, and 8 h (*n* = 12/treatment/time point). The number of bacteria adhered to the HAp discs was determined at each time point and analyzed by two-way ANOVA and Bonferroni tests. Cell-wall proteins were extracted from adhered, planktonic, and inoculum (baseline) bacteria and proteome profiles were obtained after a bottom-up proteomics approach. The number of adhered bacteria significantly increased over time, being the mean values obtained at 8 h, from highest to lowest, as follows: DR9-RR14 > statherin > RR14 = DR9-DR9 > DR9 = histatin3 > saliva (*p* < 0.05). Treatments modulated the bacterial proteome upon adherence. The findings suggested a potential use of our engineered peptide DR9-DR9 to control *S. mutans* biofilm development by reducing bacterial colonization.

## 1. Introduction

Once exposed to saliva, dental surfaces are covered by an acellular film known as the Acquired Enamel Pellicle (AEP) [1], formed by the adsorption of specific salivary proteins and peptides [2,3,4]. The peptides found in the AEP originate from the proteolysis of different salivary proteins, once secreted in the oral cavity, by salivary proteases of bacterial and human origin [5]. The AEP components act as receptors for bacterial ligands (adhesins), leading to a highly specific and irreversible attachment of bacterial cells to the dental surface. Thus, the AEP acts as a conditioning film that influences which bacteria will first adhere to the dental surfaces [6], controlling the initial steps of biofilm formation. 

Dental biofilm is a complex microbial community, developed on the tooth surface, embedded in a matrix composed of extracellular biopolymers of bacterial and host origin [7,8]. Different microorganism species coexist in the dental biofilm, keeping the physiological balance between health and disease. This homeostasis can be altered by certain environmental conditions in the oral cavity that favor the transition from healthy to pathogenic dental biofilms, such as the acidification of the biofilm fluid after the metabolism of dietary fermentable carbohydrates [9]. Acidic environments favor the dominance of acidogenic and aciduric bacteria such as *Streptococcus mutans* [10] and provoke the progressive dissolution of the mineral phase of the teeth, contributing to the development of one of the most prevalent oral diseases worldwide, namely dental caries [11].

As the AEP constituents play a key role in the bacterial colonization of dental surfaces [6], the modification of its proteinaceous components may control the adhesion of certain pathogenic microorganisms to the teeth. In this regard, it has been demonstrated that the salivary proteins statherin and histatin can reduce the adhesion of *S. mutans,* considered the most cariogenic microorganism found in the dental biofilm [12]. Moreover, salivary proteins known to exert antimicrobial functions such as cystatins, lysozyme, myeloperoxidase, and histatins were identified in the AEP [2], some of them conserving their biological activity when adsorbed onto dental surfaces [13]. Even though it is not known if histatins conserve their antimicrobial function upon adsorption, the antimicrobial activity of histatin 3 and its naturally derived 14-residue peptide known as RR14 against *S. mutans* was recently demonstrated [14].

The adaptation of salivary proteins in response to evolutionary pressure is reflected in the formation of functional complexes by certain salivary proteins once secreted into the oral cavity, protecting them against proteolysis, modulating their biological functions, and allowing their distribution throughout the oral cavity [5]. The natural existence of proteinaceous complexes displaying multiple functions served as the basis for the development of the engineered hybrid peptide DR9-RR14 [14,15,16], which has antibacterial [14] effects against *S. mutans.*

The knowledge of the functions exerted by these proteins suggests the possibility of using the naturally-occurring peptides DR9 [16,17] and RR14 [18], derived from statherin and histatin, respectively, and their engineered forms DR9-DR9 and DR9-RR14 [14], to control the caries process by reducing the adhesion of *S. mutans* to dental surfaces. Thus, the aim of this study was to assess the effects of our engineered salivary peptides on the adhesion of *S. mutans* to hydroxyapatite (HAp) and to evaluate how they modulate the bacterial proteome upon binding to each single-component pellicle-coated surface. 

## 2. Materials and Methods

### 2.1. Experimental Design

An in vitro *S. mutans* adherence model [19] was used to assess the effect of engineered salivary peptides on 1) the adherence of *S. mutans* to HAp and 2) the bacterial proteome upon binding to each single-component pellicle-coated surface. HAp discs were treated with one of the following: statherin, histatin 3, DR9, DR9-DR9, DR9-RR14, RR14, or parotid saliva (1 mg protein mL^−1^) (positive control). The coated discs were incubated with *S. mutans* UA159 for 2 h, 4 h, and 8 h (*n* = 12/treatment/time point). Adhered bacterial counts (Colony Forming Units—CFU/disc*10^5^) were determined at each time point in six discs. Proteome profiles were obtained after the mass spectrometry identification of the proteins extracted from adhered, planktonic, and inoculum (baseline) bacteria. Two independent assays per experimental condition were performed. Our hypothesis was that the engineered salivary peptides would reduce bacterial adherence, which was tested at a significant level α of 5% (two-way ANOVA and Bonferroni test).

### 2.2. Collection of the Parotid Gland Saliva

This study was approved by the Ethics Committee on Human Research at The University of Western Ontario (protocol number 16181E). Parotid gland saliva was collected as described elsewhere [19].

### 2.3. Proteins and Peptides Tested

Synthetic statherin was purchased from Peptide Protein Research Ltd. (Funtley, Hampshire, UK), while synthetic histatin 3 and peptides derived from statherin or histatin 3 were purchased from Synpeptide (Shanghai, China). All proteins and peptides used in this study are listed in Table 1. Purity (>95%) and molecular mass (Mr) from each protein and peptide were verified by high-performance liquid chromatography and mass spectrometry analysis. The isoelectric points (pIs) of statherin, histatin 3, and their peptides listed in Table 1 were calculated using the Scansite option [20]. Protein and peptide solutions were prepared in 50 mM NaCl pH 6.8 at a final concentration of 198 µM, 24 h before starting the experiment. The final concentration was checked in a UV-light spectrophotometer at a wavelength of 215 nm. 

### 2.4. Hydroxyapatite Disc Preparation

**Figure 1:**
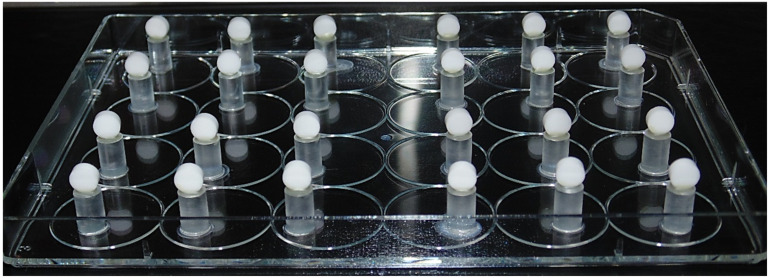
Photograph of hydroxyapatite discs assembled on a cell-culture plate lid.

Dense ceramic HAp discs with a 5 mm diameter and 2 mm thickness (Clarkson Chromatography Products Inc., South Williamsport, PA, USA) were vertically assembled in acrylic holders attached to the lid of a 24-well culture plate, leaving an area of approximately 70.7 mm^2^ exposed (Figure 1). Subsequently, the assembled lid with the HAp discs was sterilized by exposing it to ultraviolet light for 1 h.

### 2.5. Acquired Pellicle Formation

HAp discs were positioned in a 96-well culture plate and incubated for 24 h at 37 °C under constant agitation at 60 rpm/min with one of the following treatments (200 µL/well/disc): statherin, histatin 3, DR9, DR9-DR9, DR9-RR14, RR14, parotid saliva. 

### 2.6. S. mutans Inoculation and Adhesion to the AEP

After the AEP formation, the HAp discs were washed three times with 50 mM NaCl pH 6.8 and then transferred to a 24-well culture plate containing 2 mL of Tryptone Yeast Extract Broth (TYEB) supplemented with 1% glucose (*w/v*) and standard cell suspension (*S. mutans* UA159, 1 × 10^8^ CFU mL^−1^) and incubated for 2 h, 4 h, or 8 h at 37 °C and 10% CO_2_ to allow the bacteria to adhere to the HAp surface (*n* = 12/treatment/time point).

### 2.7. Quantitation of Adhered S. mutans

To determine the number of bacteria adhered to the surface of the discs at each time point, a protocol previously reported [21] was here modified. For this, a calibration curve of fluorescence intensity versus the number of viable bacteria (CFU) was created by serially diluting the standard cell suspension (*S. mutans* UA159, 1 × 10^8^ CFU mL^−1^) from pure until 1:640 in 2 mL of 1% resazurin (*v/v*) in KCl buffer (1 mM K_2_HPO_4_ pH 7.0, 0.05 M KCl, 1 mM CaCl_2_, and 0.1 mM MgCl_2_) supplemented with 0.5% glucose (*v/v*). To determine the number of viable bacteria at each dilution, three 20 μL drops of each dilution were plated in Todd Hewitt Broth (THB) Agar and incubated for 48 h at 37 °C and 10% CO_2_. The remaining suspension from each dilution was incubated for 2 h at 37 °C and the fluorescence intensity was measured with a fluorescence spectrophotometer (excitation wavelength, 560 nm; emission wavelength, 590 nm; model 650-40; Hitachi, Tokyo, Japan). At each adhesion time point, six out of twelve HAp discs per treatment group were washed three times with KCl buffer and then incubated for 2 h at 37 °C with 2 mL of 1% resazurin (*v/v*) in KCl buffer supplemented with 0.5% glucose (*v/v*). The number of viable adhered bacteria (CFU/disc) was determined after measuring the fluorescence intensity of the suspension in contact with each disc using a fluorescence spectrophotometer. 

### 2.8. Extraction of Cell Wall Proteins from S. mutans 

The remaining 6 discs per treatment were used to extract the bacterial proteins. At each time point, the corresponding HAp discs were washed three times with 0.9% NaCl (*w/v*), transferred to a microcentrifuge tube containing 1 mL of buffer (20 mM Tris-HCl pH 8.0, 10 mM MgCl_2_, 1 mM PMSF), and then sonicated at an amplitude of 10% (Sonic dismembrator, model 500, Fisher Scientific, Hampton, NH, USA) for 30 s on ice to detach the bacteria adhered to the HAp discs. The discs were carefully removed, and the bacterial suspension was centrifuged at 4000 rpm for 5 min at 4 °C. The planktonic cells in the culture medium from each of the six wells were pooled in a 15 mL tube and centrifuged at 4000 rpm for 5 min at 4 °C. The pellet having the pooled adhered or the planktonic bacteria per treatment was resuspended in 150 µL of mutanolysin mix containing 20 mM of Tris-HCl pH 8.0, 10 mM of MgCl_2_, 20% sucrose (*w/v*), 1 mM of PMSF, 100 U mL^−1^ of mutanolysin (M9901, Sigma, Saint Louis, MO, USA), and 1000 U mL^−1^ lysozyme (GTX65489, GeneTex, Irvine, CA, USA). Protein extraction was done by incubating the suspension for 2 h at 37 °C, shaking the mixture every 20 min. The extracted cell wall proteins were dialyzed to clean them up from the buffer components using tubing with a molecular weight (MW) cutoff of 1000 Da (Spectra/Por; Spectrum Laboratories Inc., Rancho Dominguez, CA, USA). Dialysis was performed for 24 h at 4 °C against 20 L of deionized water with two water volume changes. After dialysis, the protein concentration was determined by BCA assay using a spectrophotometer at a wavelength of 562 nm. A protein equivalent of 5 μg was digested with trypsin (5% trypsin—*v/v*—in 50 mM NH_4_HCO_3_, pH 7.8) for 16 h at 37 °C and purified with a C18 column for proteomic analysis. An aliquot of the bacterial suspension used to inoculate the HAp discs was processed to extract the bacterial proteins following the protocol previously described, and the obtained proteome profile was used as a baseline (time point zero). 

### 2.9. Bacterial Cell Wall Proteome Profile 

The analysis of tandem mass spectrometry was performed according to the protocol described by Siqueira and Oppenheim [4] using a Velos LTQ (Thermo-Finnigan, San Jose, CA, USA), which allows liquid chromatography to be performed in line with a capillary column C18 connected to the mass spectrometer using electrospray ionization on a test scanner in the range of *m/z* values of 390–2000 and, at the same time, performing tandem MS/MS analysis. Subsequently, the samples were diluted in a buffer containing 80% acetonitrile/19.9% H_2_O/0.1% trifluoroacetic acid (*v/v*), dried, and resuspended in 10 µL of 97.5% H_2_O/2.4% acetonitrile/0.1% formic acid (*v/v*), and were subsequently subjected to reverse-phase LC-ESI-MS/MS. The 50 μm × 10 cm reverse phase HPLC capillary column was packed in the laboratory using a Magic C18 resin 3 μm diameter and 200 Å pore size (Michrom BioResources, Auburn, CA, USA). The column was developed with a linear gradient of solvent B between 5% and 90% (0.1% formic acid in acetonitrile) (*v/v*) at a flow rate of 200 nL minute^−1^. The electrospray voltage and the ion transfer capillary temperature were 1.8 kV and 250 °C, respectively. 

The obtained MS/MS spectra were searched against *S. mutans* protein databases (Swiss Prot and TrEMBL, Swiss Institute of Bioinformatics, Geneva, Switzerland, http://ca.expasy.org/sprot/, accessed on 19 April 2019) using the SEQUEST algorithm in the Proteome Discoverer software, version 2.4 (Thermo Scientific, San Jose, CA, USA). The search results were filtered for a false discovery rate of 1%, employing a decoy search strategy utilizing a reverse database. An additional inclusion criterion for the positive identification of proteins was that the same protein passed the filter score in at least three different MS analyses from the same group in a total of three mass spectrometry analyses per group. The proteins identified specifically during the adhesion phase at each time point (2 h, 4 h, 8 h) were determined by comparison with the proteome profiles obtained from the baseline and planktonic bacteria.

### 2.10. Statistical Analysis

The normal distribution of the errors and the homogeneity of the variances were checked. Once these assumptions were checked, the number of viable bacteria adhered to HAp (CFU/disc*10^5^) was analyzed by a two-way analysis of variance (ANOVA) followed by Bonferroni test, considering the following factors: treatment at seven levels (statherin, histatin 3, DR9, DR9-DR9, DR9-RR14, RR14, parotid saliva), and time at three levels (2 h, 4 h, and 8 h). All statistical analyses were performed at a significance level α of 5%, using SPSS (IBM SPSS Statistics for Windows, Version 28.0. Armonk, NY, USA: IBM Corp.).

## 3. Results

The pIs were calculated for each peptide and protein at pH 6.8. Histatin 3 and statherin, as positively and negatively charged proteins, respectively, demonstrated pIs of 10.4 and 4.4. DR9-DR9 exhibited the lowest pI value (3.4), just below that of the natural statherin peptide DR9, whereas RR14 showed the highest value (11.0) and DR9-RR14 was intermediate (7.1). Values are provided in Table 1.

### 3.1. Quantitation of Adhered S. mutans

The statistical analysis showed that there was a statistically significant interaction between the effects of treatment and time on the number of viable bacteria adhered to HAp (CFU/disc*10^5^) (*p* < 0.0001) (Table 2). Therefore, pairwise comparisons were run for each factor under study.

**Figure 2:**
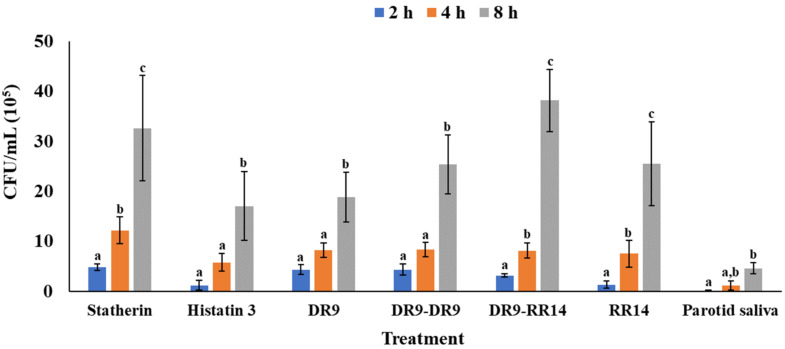
Colony-forming units (CFU/disc*10^5^) of *S. mutans* adhered to hydroxyapatite (HAp) discs within treatment according to the adhesion time. Distinct lower-case letters show significant differences among time points within each treatment (ANOVA and Bonferroni test, *p* < 0.05) (Mean ± SD; *n* = 6).

The mean values of the number of viable bacteria for each treatment at each time point are shown in Table 2. The pairwise comparison made within treatments at each time point (Figure 2) showed significant differences among groups (*p* < 0.05). As expected, the number of bacteria adhered increased over time, with the mean values obtained at 2 h being statistically different from those obtained at 4 h and 8 h within the groups statherin, DR9-RR14, and RR14, while in the remaining groups, a significant difference was only observed between 4 h and 8 h (*p* < 0.05) (Figure 2).

**Figure 3:**
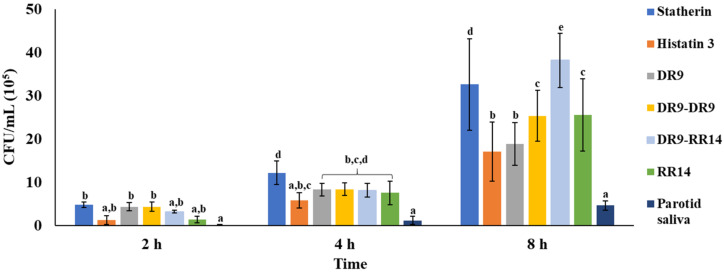
Colony-forming units (CFU/disc*10^5^) of *S. mutans* adhered to hydroxyapatite (HAp) discs at each time point according to the treatments. Distinct lower-case letters show significant differences among treatment groups at the same time point (2 h, 4 h, and 8 h) (ANOVA and Bonferroni test, *p* < 0.05) (Mean ± SD; *n* = 6).

Regarding the pairwise comparison made at each time point among treatments, a significant difference was also obtained (*p* < 0.05). Figure 3 shows that after an incubation period of 2 h, the adhesion of *S. mutans* cells was higher on the HAp discs treated with statherin, DR9, and DR9-DR9, the values of which were significantly different from the positive control, where the lowest number of adhered bacteria was detected (*p* < 0.05). When the adhesion time was prolonged to 4 h, the statherin group showed the highest number of adhered bacteria, with its value being significantly higher than those found in the histatin 3 and parotid saliva treatment groups (*p* < 0.05), and similar to the remaining groups (DR9, DR9-DR9, DR9-RR14, RR14; *p* > 0.05). At 8 h, the positive control was significantly different from all other treatment groups, being the group with the lowest number of adhered bacteria at all the time points evaluated (*p* < 0.05). According to the statistical analysis, the mean values obtained at this time point in each group were, from highest to lowest, as follows: DR9-RR14 > statherin > RR14 = DR9-DR9 > DR9 = histatin3 > parotid saliva (*p* < 0.05).

### 3.2. Cell Wall Bacterial Proteome Profiles at Baseline, and in the Adhered and Planktonic Bacteria

The list of cell wall proteins identified at baseline is shown in Table 3. Most of the proteins participate in translation and protein synthesis (63.2%), while the remaining proteins take part in carbohydrate metabolism and energy production (13.2%), and other biological processes (23.6%). The majority (87%) of the proteins identified at baseline were also found in the planktonic bacteria at the three time points evaluated (Supplementary Table S1). 

Thirty-nine out of 65 proteins were found to be expressed exclusively in the adhered bacteria (Table 4). These proteins were identified after comparing the proteome profiles obtained from the adhered *S. mutans* with the baseline (Table 3) and planktonic bacteria (Supplementary Table S1). From those proteins, the uncharacterized proteins SMU_1979c were identified only in the adhered bacteria from almost all the treatment groups and at the three time points evaluated, while the transporter PacL was found in almost all groups after 2 h and 4 h of adherence (Table 4). Only two proteins participating in glycolytic processes were identified in adhered bacteria: Eno and Pgk, Pgk being expressed in all groups after 8 h of adherence (Table 4). As for the proteins involved in bacterial adherence and biofilm formation, only GtfB and GbpB were identified at 8 h. GtfB was identified in the groups treated with statherin, RR14, and parotid saliva, while GbpB was found in DR9-RR14, RR14, and parotid saliva groups (Table 4). 

## 4. Discussion

The results of this study showed that the number of bacterial cells adhered increased over time, independently of the composition of the AEP (Table 2 and Figure 2). As expected, bacterial counts at 8 h were significantly higher, in all treatment groups, than the numbers obtained at 2 h and 4 h (Figure 2), explained by the fact that bacterial growth occurs at an exponential rate, an event successfully reproduced in the model used to perform these experiments [19]. During the eight hours of bacterial adherence, unspecific and specific mechanisms of HAp colonization may have taken place, allowing *S. mutans* cells to come into close contact with the proteins/peptides used to form the AEP [22,23]. Previous studies have shown that streptococci adhere to bare HAp through physicochemical interactions, since this substratum exhibits both anionic and cationic regions, the anionic regions being the predominant ones [24]. If the anionic cell-wall components of *S. mutans* interact directly with HAp, this attachment must be mediated by the cationic regions of the mineral. However, as negatively charged regions predominate in the HAp surface, this phenomenon is unlikely to happen due to the electrostatic repulsion between the bacteria and the mineral surface. Then, the neutralization of the net HAp surface charge mediated by the adsorption of the calcium from the culture medium onto the HAp increased the electrostatic forces between both anionic bacterial and mineral surfaces [25].

The comparison made among treatments at each time point showed that the composition of the AEP did affect the number of bacteria adhered to HAp, with the most significant differences found at 8 h (Figure 3). At this time point, the number of bacteria attached to the surface treated with statherin was significantly higher than that obtained with almost all the other groups, while similar to the engineered peptide DR9-RR14. The great number of adhered bacteria observed in the group treated with statherin suggests that this protein exposes receptor sites (cryptitopes) at the C-terminal region for oral bacteria ligands upon the dental surface, facilitating the bacterial adhesion and subsequent proliferation on the dental surface, as previously observed with other oral microorganisms [26]. As the statherin-derived peptide DR9 belongs to the N-terminal region of the protein [14,16], the absence of receptors at the C-terminal region may be the first explanation as to the lower number of bacteria found adhered to the AEP formed from the salivary peptides DR9 and DR9-DR9 (Figure 3). The second reason fewer bacteria were found adhered to the HAp discs treated with the statherin-derived peptides DR9 and DR9-DR9 is because of the acidic nature of these peptides at pH 7.0. Previous studies have shown that adsorbed acidic proteins reduce bacterial attachment by increasing the repulsive electrostatic forces between the anionic protein and the bacterial cell wall [27,28], or by competing with bacteria for HAp-binding sites [29].

Another mechanism used by the proteins/peptides to inhibit the bacterial adhesion may be the exertion of antimicrobial activity against *S. mutans*, reducing its viability once it comes into contact with the dental surface. Recently, it was demonstrated that histatin 3 and the hybrid peptide DR9-RR14 display antibacterial activity against *S. mutans* in the planktonic state [14]. However, in this study, DR9-RR14 was the one that promoted the highest bacterial adherence at 8 h in comparison with the other groups (Figure 3), suggesting that this peptide cannot exert antimicrobial activity against *S. mutans* once adsorbed onto HAp. Contrary to the reduction in bacterial attachment induced by acidic proteins, as previously discussed, basic proteins promote bacterial adherence [27,28]. This phenomenon explains why more bacteria were found attached to the HAp discs treated with RR14 than with histatin 3, since it was the molecule that exhibited the highest pI, and, therefore, the highest cationic charge at the pH used during bacterial adherence (7.0) (Table 1). As the hybrid peptide has a pI of 7.1, its charge would have been close to neutral during the adherence experiments, and no electrostatic forces mediated the attachment of *S. mutans* to HAp. In addition, the predicted secondary structure of the DR9-RR14 peptide suggests an α-helix formation in the amino acids from the 3rd to the 15th positions [30], considered a fundamental structure for the protein/mineral interaction [31,32,33]. Thus, we speculate that the remaining eight amino acids in the C-terminal region of the hybrid peptide may be forming a cryptitope that promotes specific bacterial adherence; however, the mechanism employed by DR9-RR14 to promote bacterial adherence remains to be further elucidated.

Our findings indicate that the number of bacteria adhered to the AEP formed from parotid saliva was significantly lower than all other treatment groups over time (Figure 3). In this study, the group treated with histatin 3 showed a lower bacterial adherence over time (Figure 2). This finding suggests that the inhibitory effect observed in the parotid saliva group may be due to the greater amount of this protein (and others from the histatin family) in the parotid saliva, from which the protein concentration was adjusted to 1 mg/mL. Furthermore, the saliva used may contain a concentration of histatins four times higher than that used to form the single-component AEP formed from histatin 3 (198 µM) [34], leading to the inhibition of the growth of *S. mutans* [35,36]. Moreover, the glycoproteins found in parotid saliva promote bacterial adhesion to HAp, but its adsorption onto HAp may be inhibited by the anionic phosphoproteins histatin 1 and statherin, which compete with larger salivary glycoproteins for a similar binding site upon HA surfaces [10,37]. Although the treatment of *S. mutans* with AEP-forming salivary proteins in suspension, before attaching to dental surfaces, reduces bacterial colonization [38], further studies may be needed to test if the same phenomenon occurs when our engineered salivary peptides DR9-DR9 and DR9-RR14 are used. 

Concerning the bacterial proteome analyses, the data suggest that the treatments performed to form the single-component AEP onto HAp did not modulate the bacterial proteome upon adherence, as the proteins participating in different biological processes were identified at all time points evaluated (Table 4). It is noteworthy that PacL and SMU_1979c were the only proteins expressed in all groups at the three time points evaluated (Table 4). On one hand, SMU_1979c is a protein with a conserved domain with adenine-specific DNA methylase activity [39], regulating cellular processes such as the initiation of replication, recombination, and repair. To the best of our knowledge, this is the first study in which this protein was found to be expressed exclusively by the adhered *S. mutans*, suggesting that it may play a critical role in the regulation of gene expression during the growth of adhered bacteria. Based on the functions exerted by other DNA methylases expressed by *S. mutans* (SMU_504), it can be suggested that SMU_1979c may also regulate the expression of virulence factors associated with the cariogenic potential of this bacterium [40]. Thus, further studies should be conducted to decipher the role of SMU_1979c on bacterial adherence and the cariogenicity of *S. mutans* biofilms. On the other hand, PacL, a cation-transporting P-ATPase, has been found expressed at early stages of biofilm formation [41], but, in this study, we found this protein mainly at 2 h and 4 h of *S. mutans* adherence to HAp (Table 4). This finding indicates that glucose added to supplement the culture medium acted as an environmental stressor for *S. mutans* only during the initial stages of bacterial adherence. The osmotic stress might have been reduced at 8 h by the reduction in glucose availability in the culture medium, as the bacteria used it as the main source of energy production. Regarding the expression of proteins participating in glycolytic processes, only the phosphoglycerate kinase protein Pgk was identified in all treatment groups, but only at 8 h (Table 4), confirming the active metabolism of glucose by *S. mutans* at this time point. Although we were expecting to find more proteins involved in bacterial adherence and biofilm formation at the three adherence times evaluated, only the proteins GbpB and GtfB were commonly identified at 8 h in the parotid saliva group, whereas GbpB was found at the same time point in the DR9-RR14 group (Table 4). Even though GbpB may play a role in biofilm formation [42], this protein may be mainly involved in cell wall synthesis and cell shape maintenance [42,43], explaining why GbpB was identified at baseline as well, before the inoculation of HAp discs with *S. mutans* (Table 3). In addition to GbpB, the surface protein adhesion, SpaP, was also identified at baseline (Table 3). However, SpaP mediates *S. mutans* adherence to dental surfaces by interacting specifically with salivary agglutinin [44], and, therefore, the absence of this protein in the treatment solutions may have led to the downregulation of its expression during bacterial adherence. Altogether, the results from the proteomics analysis carried out on the cell wall proteins extracted from *S. mutans* suggests that proteins other than those involved in biofilm formation may have been playing an important role in bacterial adherence onto HAp treated with our engineered salivary peptide, DR9-RR14, a matter that should be further explored. These findings also confirm that electrostatic charge interactions may be more important than specific adhesin-receptor binding when the DR9-DR9 peptide is used, as discussed above.

Regarding the methodological aspects of this study, the proposed resazurin-based method to quantify bacteria adhered to the dental surfaces is suitable to differentiate the number of adhered bacteria at different time points when the HAp surface is treated with a specific protein/peptide (Table 2). Based on the data obtained from the positive control group (Figure 3), further studies should evaluate the inhibitory effect of concentrations higher than 198 µM of our engineered peptides on the adhesion of *S. mutans* to HAp. Moreover, the observed effect on the adhesion of our engineered salivary peptides used as a single peptide solution may differ if combined with saliva, due to the possible interaction between the salivary components and our engineered peptides during the formation of the AEP, which was not simulated [45]. 

In summary, this investigation, using a single-component AEP approach and an *S. mutans* adhesion model, suggested, for the first time, the potential use of the engineered salivary peptide DR9-DR9 to control *S. mutans*’ colonization of dental surfaces.

## Figures and Tables

**Table 1:** Constructed peptides derived from statherin and histatin and their calculated isoelectric points (pI).

Peptide/Protein Name	Peptide Sequence	pI
Statherin	DSpSpEEKFLRRIGRFGYGYGPYQPVPEQPLYPQPYQPQYQQYTF	4.4
Histatin 3	DSHAKRHHGYKRKFHEKHHSHRGYRSNYLYDN	10.4
DR9	DSpSpEEKFLR	3.6
DR9-DR9	DSpSpEEKFLRDSpSpEEKFLR	3.4
DR9-RR14	DSpSpEEKFLRRKFHEKHHSHRGYR	7.1
RR14	RKFHEKHHSHRGYR	11.0

Sp represents a phosphorylated serine.

**Table 2:** Colony-forming units (CFU/disc*10^5^) of S. mutans adhered to hydroxyapatite (HAp) according to the treatments and at each time point evaluated.

Treatment		Time (h)		
	*n*	2	4	8
		Mean of CFU/Disc*10^5^ (±S.D.)		
Statherin	6	4.8 (±0.7)	12.2 (±2.7)	32.6 (±10.6)
Histatin 3	6	1.2 (±1.0)	5.8 (±1.7)	17.0 (±6.9)
DR9	6	4.3 (±1.0)	8.3 (±1.4)	18.8 (±5.0)
DR9-DR9	6	4.3 (±1.1)	8.3 (±1.4)	25.3 (±5.9)
DR9-RR14	6	3.2 (±0.3)	8.1 (±1.5)	38.1 (±6.2)
RR14	6	1.3 (±0.7)	7.5 (±2.7)	25.5 (±8.4)
Parotid saliva	6	0.0 (±0.2)	1.1 (±1.0)	4.6 (±1.1)

**Table 3:** *S. mutans* cell-wall proteome profile at baseline (time point zero).

Gene Name	Protein Name	Protein Function
*adhE*	Aldehyde-alcohol dehydrogenase	Alcohol metabolic process
ilvC *	Ketol-acid reductoisomerase (NADP(+))	Amino acid biosynthesis
gapC *	Glyceraldehyde-3-phosphate dehydrogenase	Carbohydrate metabolic process
spaP *	Cell surface antigen I/II	Cell wall antigen
hup *	DNA-binding protein HU	Chromosome condensation
eno *	Enolase	Glycolytic process
pgk *	Phosphoglycerate kinase	Glycolytic process
fbaA *	Fructose-1,6-biphosphate aldolase	Glycolytic process
gpmA *	2,3-bisphosphoglycerate-dependent phosphoglycerate mutase	Glycolytic process
*lplA*	Lipoate--protein ligase	Protein biosynthesis
groL *	60 kDa chaperonin	Protein folding
dnaK *	Chaperone protein DnaK	Protein folding
clp *	Putative Clp-like ATP-dependent protease, ATP-binding subunit	Transcription
tuf *	Elongation factor Tu	Translation
rplL *	50S ribosomal protein L7/L12	Translation
rplE *	50S ribosomal protein L5	Translation
rpsC *	30S ribosomal protein S3	Translation
rpsE *	30S ribosomal protein S5	Translation
rplK *	50S ribosomal protein L11	Translation
rpsB *	30S ribosomal protein S2	Translation
rpsJ *	30S ribosomal protein S10	Translation
rplU *	50S ribosomal protein L21	Translation
rplA *	50S ribosomal protein L1	Translation
rpsD *	30S ribosomal protein S4	Translation
*rpsG*	30S ribosomal protein S7	Translation
rplO *	50S ribosomal protein L15	Translation
rs1 *	Putative ribosomal protein S1 sequence-specific DNA-binding protein	Translation
rpsH *	30S ribosomal protein S8	Translation
rplJ *	50S ribosomal protein L10	Translation
fusA *	Elongation factor G	Translation
rplF *	50S ribosomal protein L6	Translation
rplX *	50S ribosomal protein L24	Translation
*rpsS*	30S ribosomal protein S19	Translation
tsf *	Elongation factor Ts	Translation
oppA *	Putative oligopeptide ABC transporter, substrate-binding protein OppA	Transport
livK *	Putative ABC transporter, branched chain amino acid-binding protein	Transport
SMU_1641c *	Uncharacterized protein	Uncharacterized
*SMU_63c*	Uncharacterized protein	Uncharacterized

* Represents the proteins that were commonly identified at baseline and in the planktonic bacteria.

**Table 4:** Cell wall proteins identified in the adhered *S. mutans* according to the treatments and time points evaluated.

		Time (h)																					Protein Function
		2							4							8							
	Treatment	A	B	C	D	E	F	G	A	B	C	D	E	F	G	A	B	C	D	E	F	G	
Gene Name																							
carB *						x													x				Amino acid biosynthesis
*SMU_241c*			x										x	x	x								Amino acid transport
*gbpB*																				x	x	x	Biofilm formation
gtfB *																x					x	x	EPS biosynthesis
*eno*																x				x			Glycolytic process
*pgk*														x		x	x	x	x	x	x	x	Glycolytic process
recG *										x			x										Metabolic processes
SMU_1367c *			x			x												x					Metabolic processes
*SMU_546*								x													x		Metabolic processes
*dnaK*																x			x				Protein folding
*groL*																x			x		x		Protein folding
SMU_488 *		x	x	x				x		x	x	x					x			x	x		Protein folding
SMU_1779 *		x					x	x	x				x		x								RNA methylation
Rny *		x	x					x					x				x						RNA processing
ciaR *							x						x								x	x	Transcription
*mtlR*										x					x	x				x			Transcription
*rpoC*			x										x		x			x		x	x	x	Transcription
*rplL*																x				x			Translation
pacL *				x	x	x	x	x	x	x	x		x		x	x	x						Transport
SMU_1093 *																					x	x	Transport
SMU_723 *						x		x															Transport
SMU_946 *									x														Transport
SMU_1116c *												x	x		x								Uncharacterized
SMU_1140c *													x		x								Uncharacterized
*SMU_1641c*																x			x	x	x		Uncharacterized
SMU_1979c *			x	x		x		x	x	x	x	x	x	x	x	x	x				x		Uncharacterized
SMU_2073c *						x							x		x	x				x	x	x	Uncharacterized
SMU_252 *													x		x								Uncharacterized
SMU_49 *				x																x			Uncharacterized
SMU_631 *																					x	x	Uncharacterized
*satE*																				x	x	x	Unknown

Statherin (A), Histatin 3 (B), DR9 (C), DR9-DR9 (D), DR9-RR14 (E), RR14 (F), Parotid saliva (G). * Represents the proteins that were exclusively identified in the adhered bacteria.

## Data Availability

Data is contained within the article or Supplementary Material. The data presented in this study are available in Supplementary Table S1.

## Supplementary Materials

The following supporting information can be downloaded at: https://www.mdpi.com/article/10.3390/microorganisms10020223/s1: Supplementary Table S1: List of cell wall proteins identified in the planktonic bacteria according to the treatments and at each time point evaluated.
