# Supplementary material for: Modulation of Streptococcus mutans Adherence to Hydroxyapatite by Engineered Salivary Peptides
Source: Microorganisms. 2022 Jan 20;10(2):223. doi: 10.3390/microorganisms10020223 (PMC8875007; doi:10.3390/microorganisms10020223)
Supplement: Supplementary file 1 [file microorganisms-10-00223-s001.zip › microorganisms-1511442-supplementary.pdf]

Supplementary Table S1. List of cell wall proteins identified in the planktonic bacteria according to the treatments and at each time point evaluated.

	**Treatment**																				
	**Statherin**			**Histatin 3**			**DR9**			**DR9-DR9**			**DR9-RR14**			**RR14**			**Parotid Saliva**		
**Time (h) / Gene Name**	**2**	**4**	**8**	**2**	**4**	**8**	**2**	**4**	**8**	**2**	**4**	**8**	**2**	**4**	**8**	**2**	**4**	**8**	**2**	**4**	**8**
ackA			x			x									x			x			x
acp			x			x			x			x			x			x			x
aldR			x																		
aroA			x															x			x
aroH						x															
aspB			x			x			x			x			x			x			x
bacA1					x																
clp	x	x			x		x			x									x	x	
clpC											x										
cnhA			x																		
dapH			x			x									x			x			x
dltC			x																		
dnaK	x	x	x	x	x	x	x	x	x	x	x	x	x	x	x	x		x	x	x	x
eno	x	x	x	x	x	x	x	x	x	x	x	x	x	x	x	x		x	x	x	x
fabF			x			x									x			x			x
fbaA			x			x									x			x			
flaW			x						x						x			x			
frr						x			x			x						x			x
fusA		x	x		x	x	x	x	x			x	x		x			x			x
gapC	x	x	x	x	x	x	x	x	x	x	x	x	x	x	x	x		x	x	x	x
gapN			x			x												x			x

gatA																x					
gbpB			x																		
gbpC			x			x			x						x						x
glgP									x									x			
glk			x			x						x			x			x			x
glnA			x			x			x						x			x			x
glyA			x			x															
gpmA	x	x	x		x	x		x	x		x	x			x			x	x		x
greA															x						x
groL	x	x	x	x	x	x	x	x	x	x	x	x	x	x	x	x		x	x	x	x
groS			x					x				x			x						x
hsdM					x	x		x			x			x						x	
hup	x	x	x	x	x			x			x		x	x		x		x	x	x	x
ilvC	x	x	x		x	x		x	x	x	x	x	x	x	x			x	x		x
ilvE			x			x									x			x			x
livK			x			x						x			x			x			x
mleS			x			x			x												x
mtlR		x											x			x					
nagB															x						
naoX			x			x			x						x						
oppA			x			x						x			x			x	x		x
pepN																					x
pepO									x									x			x
pfkA			x															x			x
pgi		x	x			x						x			x			x			x
pgk	x	x	x	x	x	x	x	x	x	x	x	x	x	x	x	x		x	x	x	x
pgm			x			x			x						x						
proS																		x			

psaB														x					x		
ptsG																		x			
ptsH			x																		
ptsI	x	x	x	x		x	x		x			x		x	x			x			x
purC			x																		
rmlB			x									x						x			x
rpiA			x															x			
rplA												x						x	x		
rplB																			x		
rplD																					x
rplE	x	x	x		x	x	x			x		x	x		x			x	x		x
rplF					x														x		
rplJ	x	x	x		x		x	x					x		x	x		x	x		x
rplK			x	x	x					x					x						
rplL	x	x	x		x	x	x	x	x	x	x	x	x	x	x	x		x	x	x	x
rplO					x					x			x						x		
rplV													x						x		
rplX																			x		
rpoC			x	x							x		x			x		x	x		x
rpsB					x	x	x								x						x
rpsC		x			x		x											x	x		x
rpsD					x							x						x			
rpsE	x	x		x	x		x			x			x			x		x	x		x
rpsG	x																		x		
rpsH																			x		
rpsJ	x	x		x	x		x		x	x		x	x			x		x	x		x
rpsL										x											
rpsM	x																				

rs1		x		x	x					x								x	x		x
satE			x																		
secY																x					
serC			x		x	x									x						
SMU_241c								x													
SMU_420			x																		
SMU_491					x																
SMU_743		x																			
SMU_815															x						
SMU_965			x																		
SMU_1111c			x																		
SMU_1121c			x			x									x						x
SMU_1208c											x										
SMU_1377c	x		x		x	x			x			x			x			x			x
SMU_1641c	x	x	x	x	x	x	x	x	x	x	x	x	x	x	x	x		x	x	x	x
SMU_1960c			x						x												
SMU_2084c															x						
spaP	x	x	x	x	x	x		x	x	x	x	x		x	x	x		x	x	x	x
tig			x			x												x			x
tkt												x									
tpiA		x	x		x	x	x	x	x		x	x			x	x		x			x
treA						x			x												x
trxA			x					x													
tsf		x	x		x	x		x	x	x		x			x	x		x	x	x	x
tuf	x	x	x	x	x	x	x	x	x	x	x	x	x	x	x	x		x	x	x	x
